# A Phase I trial of high dose gefitinib for patients with leptomeningeal metastases from non-small cell lung cancer

**DOI:** 10.18632/oncotarget.2886

**Published:** 2015-02-25

**Authors:** David M. Jackman, Leigh A. Cioffredi, Lorraine Jacobs, Farhana Sharmeen, Linda K. Morse, Joan Lucca, Scott R. Plotkin, Paul J. Marcoux, Michael S. Rabin, Thomas J. Lynch, Bruce E. Johnson, Santosh Kesari

**Affiliations:** ^1^ Lowe Center for Thoracic Oncology, Dana-Farber Cancer Institute, Boston, MA, USA; ^2^ Harvard Medical School, Boston, MA, USA; ^3^ Department of Neurosciences, Translational Neuro-Oncology Laboratories, Moores UCSD Cancer Center, University of California, San Diego, La Jolla CA, USA; ^4^ Georgetown University School of Medicine, Washington, DC, USA; ^5^ Eurofins Medinet B.V., Breda, The Netherlands; ^6^ Stephen E. & Catherine Pappas Center for Neuro-Oncology, Massachusetts General Hospital Cancer Center, Boston, MA, USA; ^7^ Smilow Cancer Hospital at Yale-New Haven, Yale Cancer Center, New Haven, CT, USA; ^8^ Yale University, New Haven, CT, USA

**Keywords:** non-small cell lung carcinoma, epidermal growth factor receptor, meningeal carcinomatosis, kinase inhibitors

## Abstract

**Introduction:**

There are few effective treatment options for leptomeningeal metastasis (LM) in non-small-cell lung cancer (NSCLC). This study assessed the feasibility of high-dose gefitinib in patients with LM from NSCLC harboring *EGFR* mutations or prior systemic response to EGFR-TKI.

**Methods:**

This phase I open-label trial of a novel gefitinib dosing schedule employed a 3+3 design. Eligible NSCLC patients with LM had known *EGFR* mutations and/or prior response to EGFR-TKI. Patients alternated 2 weeks of high-dose daily gefitinib (dose levels: 750 mg, 1000 mg, 1250 mg) with 2 weeks of maintenance therapy (500 mg daily). Primary endpoints were safety and toxicity. Secondary endpoints included overall survival (OS), neurological progression-free survival, radiological response, and cytological response in cerebrospinal fluid (CSF).

**Results:**

Seven patients were treated: 3 at 750 mg dose level, 4 at 1000 mg dose level. There were no DLTs at the 750 mg dose level, and one DLT (toxic epidermal necrolysis) at the 1000 mg dose level. The study was closed due to slow accrual. Median neurological PFS was 2.3months (range 1.6–4.0 months); median OS was 3.5months (range 1.6–5.1months). Though there were no radiologically documented remissions of LM disease, four patients had improvement in neurological symptoms. One patient cleared their CSF of NSCLC cells, while 2 others had decrease in malignant cells in CSF.

**Conclusion:**

Although the MTD was not defined due to slow accrual, this study provides important information about the tolerability and CSF penetration of high-dose gefitinib as a therapeutic option for modest palliation for NSCLC patients with LM and a known *EGFR* mutation.

## BACKGROUND

Leptomeningeal metastasis (LM) represents a grave development in patients with lung cancer. Lung cancer is one of the most common solid tumors to metastasize to the leptomeninges, accounting for ~13–29% of LM from solid tumors [1–4]. In the largest case series of patients with LM from solid tumors and lymphoma (*N* = 155), the patients with LM from lung cancer (*N* = 20) had a median survival of only 1.8 months, lower than any other histopathologic subgroup [2]. Moreover, therapeutic options in patients with LM are limited. While intrathecal (IT) chemotherapy has been beneficial for patients with leptomeningeal spread of lymphoma or breast cancer [5, 6], the currently available intrathecal agents (methotrexate, cytarabine, thiotepa) have limited antitumor activity in lung cancer [7]. In a randomized trial of IT methotrexate vs. IT thiotepa in patients with LM, the median survival for the 12 lung cancer patients with LM was only 8 weeks, compared with a median survival of 15 weeks for the 40 patients in the study with other non-leukemic malignancies besides lung cancer [8]. The identification of an effective treatment for leptomeningeal metastasis in lung cancer patients would be a welcome therapeutic advance.

The use of adequate concentrations of an epidermal growth factor receptor tyrosine kinase inhibitor (EGFR-TKI) may be one therapeutic possibility. In patients whose tumor harbors a sensitizing *EGFR* mutation, systemic response rates to therapy with gefitinib or erlotinib range from 60–90% [9–13]. Moreover, the ability to cause regression of brain metastases in patients with non-small cell lung cancer and *EGFR* mutations has been well described [14–16], suggesting that the agent does have adequate penetration into the CNS to have antitumor activity.

The development of subsequent LM in patients on treatment with an EGFR-TKI is felt to be a pharmacokinetic failure, wherein the penetration of EGFR-TKI into the cerebrospinal fluid is inadequate to suppress leptomeningeal tumor involvement. In a previously published case report, members of our research group had used high doses of gefitinib when it was still approved for use in the United States to treat such a NSCLC patient with leptomeningeal metastases and a known somatic exon 19 deletion mutation in his tumor EGFR [17]. Gefitinib as a single agent was increased from 500 mg up to 1250 mg per day over the course of two months. Over that time, the central nervous system (CNS) symptoms improved, and cytology from his cerebrospinal fluid (CSF) no longer showed evidence of lung cancer cells. With the use of increased dosages of gefitinib, CSF concentrations of gefitinib were over 40 nM, exceeding the *in vitro* IC50 in H3255 and DFCILU-011, two NSCLC cell lines with sensitizing mutations in the *EGFR* [18, 19]. At the time of death, port-mortem studies in this patient as well as in another patient who had developed CNS progression after an initial systemic response to gefitinib showed the development of the T790M resistance mutation in systemic sites but no such resistance mutation in the CNS metastases [17, 20].

Based on that experience, we postulated that pulsed doses of a higher dose of gefitinib might be an effective therapy for leptomeningeal metastases. We have conducted a prospective phase I trial of high-dose gefitinib therapy for LM in NSCLC patients with known *EGFR* mutations and/or a prior response to an EGFR-TKI. Based on the original gefitinib phase I studies of continuous daily dosing [21–24], doses up to 600 mg/day were generally well tolerated, without dose limiting toxicities. While grade 3 and 4 toxicities did increase at doses ≥ 700 mg/d, the majority of adverse effects were reversible with dose reduction or cessation. Moreover, in the two studies that included planned dose escalation to 1000 mg/d, doses of 1000 mg/d were administered to 17 patients [21, 25]. Eight of these patients suffered dose-limiting toxicities: all eight experienced grade 3 diarrhea, with 3 cases of grade 3 rash. However, there were no grade 4 toxicities reported at the 1,000 mg/d dose level.

Because the time to peak plasma levels for gefitinib is 7–10 days [26], we had treated our previously reported patient with an alternation of 14 days of increased drug dosage followed by 14 days of a 500 mg daily maintenance dose. This allows for 4–7 days of peak plasma levels at the higher dose, while trying to prevent or minimize any dose-related toxicity. By alternating periods of high dose and periods of maintenance therapy in our previously described patient, we had been able to administer gefitinib in doses as high as 1250 mg daily, achieving therapeutic benefit while minimizing somnolence and other toxicities that were observed in previous phase I studies [17]. Therefore, we embarked on a phase I study employing this strategy in NSCLC patients with LM and either a known sensitizing *EGFR* mutation and/or a history of prior response to an EGFR-TKI.

## RESULTS

### Patient characteristics

Between May 2006 and July 2008, seven patients with leptomeningeal metastases caused by NSCLC were enrolled and treated with high-dose gefitinib (Table 1). Six of the seven patients harbored a known sensitizing mutation in their tumor *EGFR* (five exon 19 deletions, 1 L858R mutation); a seventh patient had insufficient tissue for *EGFR* mutation analysis but was eligible for enrollment based on a radiographic response to prior therapy with an EGFR-TKI; she had received a total of 20 months of vandetanib before developing leptomeningeal metastases as her primary site of metastasis. All patients had previously been treated with an EGFR-TKI for a median of 15.0 months (range 6.7 – 20.4 months) prior to the diagnosis of leptomeningeal metastases. Of the 6 patients who had evaluable disease outside the central nervous system at the start of their prior EGFR-TKI therapy, 5 had a partial response, while the sixth had stable disease with a minor response. One patient whose primary tumor harbored an exon 19 deletion had received a prior EGFR-TKI as maintenance therapy in the setting of having no evaluable disease; therefore, it was impossible to assess response to prior EGFR-TKI in that patient.

**Table 1:** Baseline demographic and disease characteristics (*n* = 7)

Median age, years (range)	51 (46 – 72)
Race, n (%)	
White	6 (86%)
Asian	1 (14%)
Sex, n (%)	
Female	5 (71%)
Male	2 (29%)
Histology, n (%)	
Adenocarcinoma	4 (57%)
Non-small cell lung cancer, not otherwise specified	3 (43%)
Smoking status, n (%)	
Never smoker	2 (29%)
Former smoker, ≤ 10 pack years	3 (43%)
Former smoker, > 10 pack years	2 (29%)
ECOG performance status at enrollment	
1	3 (43%)
2	2 (29%)
3	2 (29%)
Brain metastases at the time of initial diagnosis, n (%)	3 (43%)
Prior whole brain radiation therapy for brain metastases, n (%)	6 (86%)
Prior systemic therapy for CNS disease, n (%)	2 (29%)
*EGFR* mutation status	
Exon 19 deletion	5 (71%)
L858R mutation	1 (14%)
Not tested	1 (14%)
Prior EGFR-TKI received	
Erlotinib only	2 (29%)
Gefitinib only	2 (29%)
Gefitinib, then erlotinib	1 (14%)
Erlotinib, then HKI-272	1 (14%)
Vandetanib plus docetaxel	1 (14%)
Prior systemic response to EGFR-TKI	
Complete response	0
Partial response	5 (71%)
Stable disease	1 (14%)
Progressive disease	0
Not evaluable	1 (14%)

Three patients had brain metastases at the time of diagnosis. All three successfully underwent cranial irradiation for these brain metastases prior to the diagnosis of LM, with radiation completed 6.2, 17.4, and 23.2 months prior to development of LM. Three other patients were treated with cranial irradiation at the time of diagnosis of LM, completing cranial irradation 2 weeks – 4 months prior to the start of high-dose gefitinib as part of the therapeutic protocol. The seventh patient did not receive whole brain radiation therapy prior to enrollment on this trial, as she did not have CNS metastases at diagnosis and subsequently developed leptomeningeal metastases without parenchymal brain metastases. There was no clear correlation between the administration or timing of whole brain radiation therapy and outcomes to high-dose gefitinib.

### Adverse events and determination of a maximum tolerated dose

All enrolled patients were assessed for toxicities (Table 2). Diarrhea was the most common drug-related toxicity (5/7), with one case of grade 3 toxicity. There were no dose-limiting toxicities (DLTs) in the gefitinib 750 mg cohort. There was one DLT in the 1000 mg cohort: after completing one cycle of therapy, a patient with a pre-existing history of Sjogren's syndrome was found to have disease progression in the CNS and systemically. After stopping the medication for a few days, she subsequently developed worsening rash that evolved into a grade 4 toxic epidermal necrolysis; of note, this patient had the highest measured steady-state plasma gefitinib concentrations (5094 nM) on this trial. She died of systemic lung cancer progression 3 weeks later.

**Table 2:** Drug-related toxicities

	750 mg (*N* = 3)		1000 mg (*N* = 4)	
Toxicity	Any grade	Grade ≥ 3	Any grade	Grade ≥ 3
Rash	2	0	3	1
Diarrhea	3	0	2	1
Fatigue	2	0	2	0
Nausea/vomiting	1	0	1	1
Elevated hepatic transaminase	0	0	2	0
Elevated alkaline phosphatase	1	0	2	0
Elevated lipase	0	0	1	0

In addition to the drug-related toxicities listed, 3 patients developed pulmonary emboli, though these were felt to be more likely related to their underlying malignancy and overall debility than to the drug. As increasing safety data for the concurrent use of low molecular weight heparin (LMWH) and gefitinib became available, the prior restriction on anticoagulation was amended to allow the use of LMWH for deep vein thrombosis or pulmonary embolism. Subsequently, one patient died after a mechanical fall that resulted in a subdural hematoma while on enoxaparin anticoagulation for a pulmonary embolism that developed 13 days into his first cycle of high dose gefitinib.

On the basis of a DLT at the 1000 mg dose level, that cohort was expanded from 3 to a planned 6 patients. However, due to slow accrual, only 4 patients were enrolled on the 1000 mg dose level, and the trial was subsequently closed to new accrual, despite an absence of additional DLT's. On this basis, while a maximum tolerated dose (MTD) for this gefitinib schedule of administration was not formally defined, the 750 mg dose level was well tolerated, without DLT.

### Efficacy

Clinical Improvement: All enrolled patients (*n* = 7) were assessed for clinical benefit. Four patients – 2 from the 750 mg dose level and 2 from the 1000 mg dose level – experienced marked clinical improvements in neurological signs and symptoms with the introduction of high-dose gefitinib. These improvements in neurological examinations were assessed by thorough neurological exams performed by neuro-oncologists as specified by the clinical protocol. As objective parameters of clinical neurologic progression were defined by the protocol (see *Treatments and Assessments,* above), the protocol also prospectively defined objective resolution of previous neurologic deficits that resulted in improvements in clinical function and performance status as constituting a clinical neurologic improvement. Three of these four patients had exon 19 deletions in their tumor *EGFR*. The fourth did not have sufficient tissue for *EGFR* mutation testing.

Two patients had received prior temozolomide for their leptomeningeal metastases, for a duration of 1 – 2 cycles. Both patients had undergone whole brain radiation therapy for brain metastases that had been present at the time of their initial lung cancer diagnosis. While both of these patients developed progression of their central nervous system disease and related symptoms while on temozolomide, they each achieved clinical improvements in symptoms related to leptomeningeal metastases while being treated with high-dose gefitinib.

Cytological and Radiological Response: One patient treated with 1000 mg of gefitinib daily had complete clearance of lung cancer cells from the CSF after 2 months of therapy as assessed by both cytology in the hematology laboratory as well as in the cytopathology laboratory. Two other patients in the trial, one at the 750 mg dose level and the other at 1000 mg dose level, had some evidence of cytological clearance of malignant cells from the cerebrospinal fluid: in both of these patients, one of two independently assessed CSF samples showed no evidence of malignancy, while the other sample in each case showed only a few residual tumor cells (Table 3). These three patients were among the four who had improvements in clinical findings and symptoms. None of the seven patients in the trial showed evidence of radiological resolution of their leptomeningeal metastases on MRI of the brain and spine.

**Table 3:** Gefitinib, steady-state CSF and plasma concentrations, and clinical outcomes

Patient	Gefitinib dose (mg)	Pretreatment *EGFR* genotype	Steady-state gefitinib concentra tion, CSF (nM)	Steady-state gefitinib concentration, plasma (nM)	Clinical improve ment	CSF cytology clearance	Radiological improve ment	CNS progression-free survival (months)	Survival (months)
1	750	Exon 19 deletion	14.7 – 17.1	1362.8 – 1835.4	Yes	Partial	No	2.7	3.9
2	750	Not done	48.8 – 54.0	1345.9	Yes	No	No	1.9	1.9
3	750	L858R	41.5	4993.0	No	No	No	1.6	1.6
4	1000	Exon 19 deletion	57.5	5094.4	No	No	No	1.0	1.6
5	1000	Exon 19 deletion	93.6 – 143.1	4090.5	Yes	Partial	No	2.7	3.5
6	1000	Exon 19 deletion	17.3 – 21.6	1552.3 – 1599.6	Yes	Yes	No	2.3	5.1
7	1000	Exon 19 deletion	21.3 – 21.8	2105 – 2935.9	No	No	No	4.0	4.0

Progression-free and overall survival: The median time to neurological progression-free survival from the time of enrollment was 2.3 months, with a range of 1.6 – 4.0 months. The median overall survival from the time of trial enrollment was 3.5 months, with a range of 1.6 – 5.1 months.

Steady-state gefitinib concentrations: In patients treated at the 750 mg dose level, the plasma gefitinib steady-state concentrations ranged from 1345.8 – 4993.0 nM, while CSF gefitinib concentrations ranged from 14.7 – 54.0 nM. At the 1000 mg dose level, plasma gefitinib steady-state concentrations ranged from 1552.3 – 5094.4 nM, while CSF concentrations ranged from 17.3 – 143.1 nM. There was no obvious correlation between oral dose, plasma concentration, or CSF concentration within the two studied dose levels. Moreover, there was no correlation between the CSF steady-state concentration achieved and clinical outcome: the subjects who achieved clinical improvements or cytological responses included patients at both the low and high end of the gefitinib CSF concentration spectrum. However, interpretation of response is limited since 3 patients never achieved the 40 nM target in CSF and 2 were borderline.

## DISCUSSION

Leptomeningeal metastasis often represents a grave finding in patients with lung cancer, with no consistently effective treatment options available. Moreover, this problem may become increasingly common in patients with *EGFR* mutations who are being treated with an EGFR-TKI [31]. First, these patients tend to have longer overall survival compared with their *EGFR* wild-type counterparts, giving them a longer time over which to develop metastases to the central nervous system: the median survival for patients with sensitizing *EGFR* mutations who are initially treated with an EGFR-TKI is over 24 months, while overall survival in wild-type patients has generally been less than a year [13, 32]. Moreover, there is evidence to suggest a trend towards an increased risk of LM for patients being treated with an EGFR-TKI. In a retrospective study comparing the risk of CNS progression in patients treated with a first-line EGFR-TKI vs. chemotherapy, the actuarial risk of LM in the EGFR-TKI group was 10% at 24 months, compared with 6% for the chemotherapy group [33]. A separate retrospective case series found that 9% of patients with *EGFR* mutant tumors and brain metastases would go on to develop leptomeningeal metastases [34]. Therefore, while this may seem a narrow population, any potential therapy options for this patient cohort would be an important advance.

In this trial, we evaluated the use of high-dose gefitinib, given daily for a 2-week period and then followed by a 2-week period of lower dose therapy at 500 mg/day. A maximum tolerated dose (MTD) for this gefitinib schedule of administration was not completely defined due to slow trial accrual; the narrow eligibility requirements of development of leptomeningeal metastasis specifically in patients with known *EGFR* mutations limited the available recruitment pool, but pharmacokinetic and safety data has been generated. First, while slow accrual prevented formal definition of the MTD for gefitinib when given in alternating fashion (2 weeks high-dose, 2 weeks lower dose), the 750 mg dose level was well-tolerated without any dose limiting toxicities, and objective improvements were seen at that dose level. Second, at both the 750 mg and 1000 mg dose level, there was consistent penetration of gefitinib into the CSF in concentrations of 14.7 – 143.1 nM, approximating the known IC50 (40 nM) for gefitinib in *EGFR*-mutant cell lines [35]. While a dose-limiting toxicity did arise in one patient of the four treated at the 1000 mg dose level, further expansion of the cohort was thwarted by slow accrual. In future multiple sampling in each patient would add value in understanding any time dependent changes in PK profiles. As well time off EGFR TKI for systemic disease was not captured and may be an important variable in assess response in future studies.

Finally, four of seven patients had improvements in clinical symptoms on therapy, with three patients showing reductions in malignant cells in the CSF. While improvements in survival were limited, the overall median survival in this trial (3.5 months) is not as dismal as those outcomes reported in prior series of patients with LM from lung cancer [8]. However, the small sample size limits broad generalization and more studies are needed to fully assess the value of alternative dosing regimens.

In the absence of other effective treatment options for LM, it is reasonable for physicians in countries where gefitinib is clinically available to consider the use of high dose gefitinib at 750 mg daily for 14 days followed by 500 mg for 14 days for NSCLC patients with LM and a known sensitizing *EGFR* mutation. Prior postmortem examinations suggest that CNS metastases in these patients will often remain sensitive to EGFR-TKI therapy [17, 20], and this study demonstrates a consistent ability to achieve adequate gefitinib CSF concentrations with this dose and schedule. In general, the current study and other published reports [36] suggest that the strategy of high doses of EGFR-TKI's like gefitinib and erlotinib can achieve sufficient CSF concentrations to effectively treat the otherwise ominous development of leptomeningeal metastases. In places where gefitinib is not commercially available, pulsed high doses of erlotinib have been studied clinically and might be an alternative consideration [36].

As *EGFR* mutation testing becomes more widely employed and more mutation-positive patients are identified and treated with EGFR-TKI's, leptomeningeal metastases in this population may become a more significant clinical problem. Going forward, it will be interesting to determine whether response of leptomeningeal metastases may be more related to peak rather than steady-state concentrations, area under the curve, or specific genotype/phenotype of cells in CSF compartment. Resistance mechanisms may arise including new mutations or clonal selection to name a few. Additional studies of these strategies to combat or prevent the development of central nervous system disease in patients with initial sensitizing mutations in their tumor *EGFR* should be considered.

## PATIENTS AND METHODS

### Patient selection

This trial was registered with the National Cancer Institute (clinical trial # NCT00372515). Eligible patients were ≥ 18 years of age with histologically or cytologically confirmed non-small-cell lung cancer; a previously documented somatic *EGFR* mutation or documented partial or complete response to prior therapy with an EGFR-TKI; ECOG performance status 0–3; and cytologically confirmed leptomeningeal metastases. Other eligibility criteria included white blood cell count ≥ 3,000/mL; hemoglobin ≥ 9.0 g/dL; platelet count ≥ 80,000/mL; creatinine ≤ 1.5 mg/dL; AST, ALT, and total bilirubin ≤ 1.5 times the institutional upper limit of normal; international normalized ratio ≤ 1.5; and activated thromboplastin time < 50 seconds. At least 2 weeks must have passed since prior radiation therapy, and at least 3 weeks must have passed since prior major surgery. Patients were excluded for a history of prior gefitinib- or erlotinib-related toxicity that had led to a dose reduction or interruption; uncontrolled brain metastases that were associated with mass effect that would contraindicate lumbar puncture; history of other malignancy within the past five years, except for definitively treated non-melanomatous skin cancers or carcinoma in situ of the uterine cervix; a gastrointestinal disorder that might compromise absorption of gefitinib; any condition requiring concurrent or ongoing use of anticoagulation; pregnancy or breastfeeding; or any severe or unstable medical condition.

Patients were enrolled and treated at the Dana-Farber Cancer Institute and Massachusetts General Hospital in Boston, MA. The study protocol was approved by the Dana-Farber/Harvard Cancer Center institutional review board, and all patients gave written informed consent. This study was conducted according to the Declaration of Helsinki and its amendments.

### Study design

This was an open-label, dose-escalation study to evaluate the safety and feasibility of high dose gefitinib in the treatment of leptomeningeal metastases NSCLC in patients with known or suspected *EGFR* mutations. The study followed a standard 3+3 design, with three preplanned dose levels (750 mg, 1000 mg, and 1250 mg). The decision for dose escalation was made after three to six patients in a cohort were evaluated for adverse events after completing one 28-day cycle of daily gefitinib. A dose-limiting toxicity was defined as any gefitinib-related grade 3, 4, or 5 toxicity as defined by the National Cancer Institute Common Toxicity Criteria (NCI-CTC) version 3.0, with the following exceptions: grade 3 diarrhea, grade 3 or 4 rash, or grade 3 or 4 elevations in hepatic transaminases would not be considered dose-limiting toxicities if they improved to ≤ grade 2 after holding therapy for up to 14 days.

### Treatment plan and assessments

Previous phase I trials of gefitinib monotherapy had established a maximum tolerated dose (MTD) of 600–700 mg with continuous daily dosing. [21–24]. In order to deliver sufficiently high doses of gefitinib without risking severe somnolence or other dose-limiting toxicity, patients were treated for 14 days at one of three planned daily dose levels of high-dose gefitinib (750 mg, 1000 mg, or 1250 mg) followed by 14 days of therapy at a “maintenance” dose of 500 mg daily, a dose previously identified as safe and employed in phase II trials [27, 28]. This 28-day period was considered one cycle of treatment. Subjects kept a pill diary, which was reviewed by study staff and compared to the remaining pill count every 14 days.

Subjects underwent a history, physical examination, and standard laboratory studies (complete blood count, chemistries, liver and renal function tests, and coagulation studies) prior to starting therapy and at the completion of both the high-dose and maintenance periods of each treatment cycle. In addition, patients underwent a detailed neurological examination by a neuro-oncologist to document any neurological deficits on days 1 and 14 (i.e., at the completion of 2 weeks of high-dose therapy) of each treatment cycle. As in other previously published clinical trials for leptomeningeal metastasis, this trial defined neurological improvement/remission as distinct neurological improvement of at least one symptom or sign, without deterioration of other neurological symptoms/signs. Neurological stable disease was defined as having no distinct change of existing neurological symptoms/signs, while progression was defined as deterioration of symptoms/signs or appearance of new neurological symptoms/signs [29]. Specific attention was paid to any new or worsening neurological deficit that was marked enough to interfere with activities of daily living (ADLs), though less severe but persistent neurological deficits were also felt to be clinically meaningful. Changes in steriod dosing were noted and accounted for in the neurological response assessment by the neuro-oncologist.

Prior to starting therapy and at day 14 of each cycle, investigators collected both blood and CSF for determination of steady-state gefitinib concentrations (see below). The collected CSF was also assessed for the presence of malignant cells. CSF cytology samples from each time point were sent for independent review to both the cytology laboratory and the hematology laboratory. A patient was considered to have cleared their CSF of malignant cells if independent reviews by cytologists in both laboratories were deemed negative for malignancy and at least 2 separate samples.

Radiographic assessments were performed at baseline and at the completion of every 28-day treatment cycle and reviewed by the treating physician and a neuroradiologist. Required scans included computed tomography of the chest and abdomen, as well as magnetic resonance imaging of both the brain and full spine to assess the extent of leptomeningeal disease. While measurable disease was assessed using Response Evaluation Criteria in Solid Tumors (RECIST), version 1.0, leptomeningeal metastases is not considered a measurable lesion. Moreover, a prior autopsy study has shown that subtle changes in MRI findings might not accurately represent changes in actual degree of leptomeningeal tumor burden [30]. As such, the current trial stipulated complete resolution of a previously noted area of leptomeningeal metastasis on MRI to meet the prospective requirement for radiologic improvement. As well, we also separately assessed clinical response.

### Steady-state gefitinib concentrations

Plasma and CSF were collected on day 14 of each treatment cycle, following the completion of the high-dose portion of gefitinib therapy. Samples were drawn between 4–6 hours after the administration of gefitinib. Samples were labeled and stored at – 20°C. CSF and plasma gefitinib steady state concentrations were determined at Eurofins Medinet B.V. (Breda, the Netherlands) using previously described methods of liquid-liquid extraction and high-performance liquid chromatography with mass spectrometric detection, though at neutral pH [22].

### Statistical analysis

Survival and neurological progression-free survival were measured from the start of therapy. Neurological progression-free survival was defined as the period from study registration until the development of either death, clinical neurological progression as determined by neurological assessment and examination by a neuro-oncologist, or radiological progression as noted on brain or spine MRI.
